# Early supplementation with live combined *Bacillus subtilis* and *Enterococcus faecium*: association with feeding intolerance and gut microbiota composition in antibiotic-exposed preterm infants

**DOI:** 10.3389/fcimb.2026.1826157

**Published:** 2026-05-19

**Authors:** Chaohui Ye, Li Li, Hongming Zhang, Qin Lyu, Yinquan Xu, Yanhong Li

**Affiliations:** 1 Clinical Trial Institution, Women and Children’s Hospital of Ningbo University, Ningbo, Zhejiang, China; 2 Department of Neonatal Intensive Care Unit, Women and Children’s Hospital of Ningbo University, Ningbo, Zhejiang, China; 3 School of Bioengineering, East China University of Science and Technology, Shanghai, China

**Keywords:** antibiotic exposure, feeding intolerance, gut microbiota, preterm infants, probiotics

## Abstract

**Background:**

Early antibiotic exposure frequently induces gut dysbiosis in preterm infants. Although probiotics, such as the live combined preparation of *Bacillus subtilis* and *Enterococcus faecium* (LCBE), may mitigate this disruption, high-quality clinical evidence in this high-risk population remains limited. This prospective observational study investigated the association of early LCBE supplementation with the incidence of feeding intolerance (FI) and gut microbiota composition in antibiotic-exposed preterm infants.

**Methods:**

Seventy-nine antibiotic-exposed preterm infants admitted to the neonatal intensive care unit (NICU) between February 2020 and April 2022 were enrolled and divided into two groups based on clinical LCBE administration: the probiotic group (n = 41) and the non-probiotic group (n = 38). Clinical data were collected prospectively. Gut microbiota composition was analyzed via 16S rRNA gene sequencing of 274 fecal samples (144 from the probiotic group, 130 from the non-probiotic group).

**Results:**

LCBE supplementation was associated with a significantly lower incidence of FI (17.07% vs. 44.74%; adjusted OR = 0.156, 95% CI: 0.050–0.491, *p* = 0.001). Notably, this protective association became evident only during the post-supplementation period (postnatal days 21–28), suggesting a delayed but sustained effect. The intervention was well-tolerated, with no treatment-related adverse events reported. In infants receiving LCBE, we observed suppression of potentially pathogenic bacteria, including *Enterococcus* and *Klebsiella*, alongside the preservation of beneficial genera such as *Bifidobacterium* and *Lactobacillus*. These microbial shifts were accompanied by a more stable and mature gut microbiota profile.

**Conclusions:**

In antibiotic-exposed preterm infants, early LCBE supplementation was associated with a significant reduction in the incidence of feeding intolerance. This clinical benefit was accompanied by favorable gut microbiota modulation, characterized by the suppression of opportunistic pathogen overgrowth and the preservation of beneficial commensal taxa. These findings support the potential role of LCBE as a safe and well-tolerated adjunctive intervention in this vulnerable population, offering practical insights for neonatologists.

## 1 Introduction

The establishment of the neonatal gut microbiota is a complex process pivotal to host health and disease susceptibility, as it mediates immune system development, nutrient digestion, and intestinal barrier integrity (Tamburini et al., 2016; Tanaka and Nakayama, 2017; Iliev et al., 2025). Compared with term infants, preterm infants exhibit marked physiological immaturity and distinct feeding patterns. Additionally, the majority are delivered via cesarean section, which limits their exposure to the maternal vaginal microbiota. These factors lead to delayed gut microbial colonization and maturation, reduced bacterial diversity, and increased pathogenic colonization (Chen and Shi, 2023), predisposing this population to a high incidence of gastrointestinal complications closely associated with microbiota dysregulation (DeWeerdt, 2018; Wolter et al., 2021; Chae et al., 2024).

Among these complications, feeding intolerance (FI) is one of the most common gastrointestinal problems in preterm infants, characterized by symptoms such as gastric residuals, abdominal distension, and vomiting, which lead to interruption or difficulty in advancing enteral feeding (Evidence-Based Medicine Group, 2020; Soderquist Kruth et al., 2025). Beyond its immediate impact on nutritional intake and growth, FI significantly prolongs hospital stay and, more critically, serves as an important precursor to necrotizing enterocolitis (NEC) (Alqahtani, 2025). The clinical management of FI is particularly challenging in preterm infants who require antibiotic therapy, as antibiotic exposure further exacerbates the underlying dysbiosis, which in turn impairs intestinal homeostasis and increases the risk of gastrointestinal adverse events (Greenwood et al., 2014; Ruiz et al., 2017). For instance, Tanaka et al. reported that early antibiotic exposure disrupts gut microbiota colonization and diversity in newborns, with a particularly marked impact on *Bifidobacterium* (Tanaka et al., 2009). Fouhy et al. showed that concurrent administration of ampicillin and gentamicin significantly impaired early intestinal microbiota development, leading to reduced abundance of *Bifidobacterium* and *Lactobacillus* in antibiotic-treated infants (Fouhy et al., 2012). Importantly, these commensal genera (such as *Bifidobacterium* and *Lactobacillus*) collectively play a critical role in maintaining neonatal intestinal health by promoting intestinal barrier maturation, producing short-chain fatty acids, modulating mucosal immunity, and providing colonization resistance against pathogenic bacteria (Dai et al., 2025; Erickson et al., 2025). The depletion of these key commensals directly impairs intestinal function and explains why feeding intolerance is particularly difficult to manage in antibiotic-exposed preterm infants.

In recent years, probiotics have garnered increasing attention for their gut-protective effects, which include enhancing intestinal barrier function, stabilizing the gut microbiome, inhibiting pathogen adhesion, and reducing intestinal permeability (Fukuda et al., 2011; Agus et al., 2018; Wang et al., 2023; Dargenio et al., 2024). Notably, Nobel et al. confirmed that probiotics can effectively ameliorate antibiotic-associated dysbiosis and related gastrointestinal adverse events (Nobel et al., 2015). Extending these findings to preterm infants, Kiu et al. demonstrated that probiotic supplementation could reduce the burden of antibiotic resistance genes and multidrug-resistant pathogens while restoring an age-appropriate microbiota—findings directly relevant to our study cohort (Kiu et al., 2025). In intervention studies targeting FI in preterm infants, probiotics have shown potential for improving gastrointestinal function and promoting enteral feeding (Soderquist Kruth et al., 2025), although efficacy varies among specific strains, highlighting the need for more high-quality clinical evidence. The live combined *Bacillus subtilis* and *Enterococcus faecium* (LCBE) formulation at the core of this study is well-documented to suppress intestinal pathogens and modulate the gut microbiota (Tompkins et al., 2010; Huang et al., 2021; Pi et al., 2022), with complementary and synergistic effects: *B. subtilis*, a facultative anaerobe, reduces intestinal oxygen tension following colonization, thereby supporting the growth of beneficial anaerobes (e.g., *Bifidobacterium*, *Lactobacillus*) and facilitating microbiota recovery, while *E. faecium* reinforces intestinal homeostasis and inhibits pathogen colonization.

Currently, LCBE is a clinically approved probiotic for intestinal microecological regulation and is hypothesized to be a promising adjunctive therapy for antibiotic-exposed preterm infants by restoring gut microbiota homeostasis and mitigating antibiotic-related gastrointestinal adverse events. However, high-quality clinical evidence supporting its use in this high-risk population remains extremely scarce. To date, only one study has assessed its efficacy in pediatric diarrhea (Rui and Ma, 2020), with no published data on its effects on antibiotic-induced gut dysbiosis and FI in preterm infants. This study aimed to assess the association of early LCBE supplementation with FI and gut microbiota composition in antibiotic-exposed preterm infants in a real-world setting and to explore the temporal dynamics of these associations, particularly whether the effects of LCBE persisted after its cessation. Our findings may inform evidence-based LCBE use and guide clinical practice in this vulnerable population.

## 2 Materials and methods

### 2.1 Study population and design

This prospective observational study enrolled consecutive preterm infants admitted to the neonatal intensive care unit (NICU) of the Women and Children’s Hospital of Ningbo University between February 2020 and April 2022. The predefined observation window was the first 28 postnatal days, with all clinical outcome assessments and fecal sample collections completed within this timeframe.

The final clinical analysis cohort included 79 eligible infants, divided into two groups based on clinical LCBE administration (Combined Bacillus subtilis and Enterococcus faecium Granules with Multivitamins, Live; Hanmi Pharmaceutical, China): the probiotic group (n = 41) and the non-probiotic group (n = 38).

Inclusion criteria were: (i) gestational age < 36 + 0 weeks and birth weight < 2500 g; (ii) early-onset infection or risk factors for perinatal infection, with empirical penicillin therapy initiated within 24 hours after birth and administered for 7 ± 1 consecutive days; (iii) exclusive feeding with standard preterm formula, with no supplementation with commercial human milk oligosaccharides. Exclusion criteria were: (i) severe gastrointestinal malformations or major congenital anomalies; (ii) severe intraventricular hemorrhage (grade 3 or higher) or moderate-to-severe hypoxic-ischemic encephalopathy (Sarnat and Sarnat, 1976; Papile et al., 1978); (iii) severe birth asphyxia or coagulopathy; and (iv) events occurring during the study period, including abdominal surgery, contraindications to enteral feeding, or the need for escalated antibiotic therapy due to disease progression.

All infants received standardized care, including individualized nutritional and respiratory support based on clinical severity. Comorbidities were managed in accordance with the Guidelines for the Management of Preterm Infants (Editorial Board of Chinese Journal of and Group of Neonatology, 2006). Enteral feeding advancement followed a unified standardized unit protocol, with daily volume increases determined by objective clinical tolerance criteria. No protocol deviations were permitted.

The dosage and duration of LCBE administration were determined according to the product instructions and local NICU standard protocols. The probiotic group received one sachet (1 g) daily for 14 consecutive days, with supplementation initiated between postnatal days 4 and 6. Each sachet contained 37.5 mg of live bacterial lyophilized powder, providing a total of 1.5×10^8^ colony-forming units (CFU) of viable microorganisms, consisting of *Enterococcus faecium* (1.35×10^8^ CFU) and *Bacillus subtilis* (1.5×10^7^ CFU). LCBE granules were dissolved in warm standard preterm formula (< 40 °C) and administered at least 2 hours apart from antibiotics to avoid probiotic inactivation. Infants in the non-probiotic group did not receive LCBE or any other probiotics during the study period.

**Figure 1:**
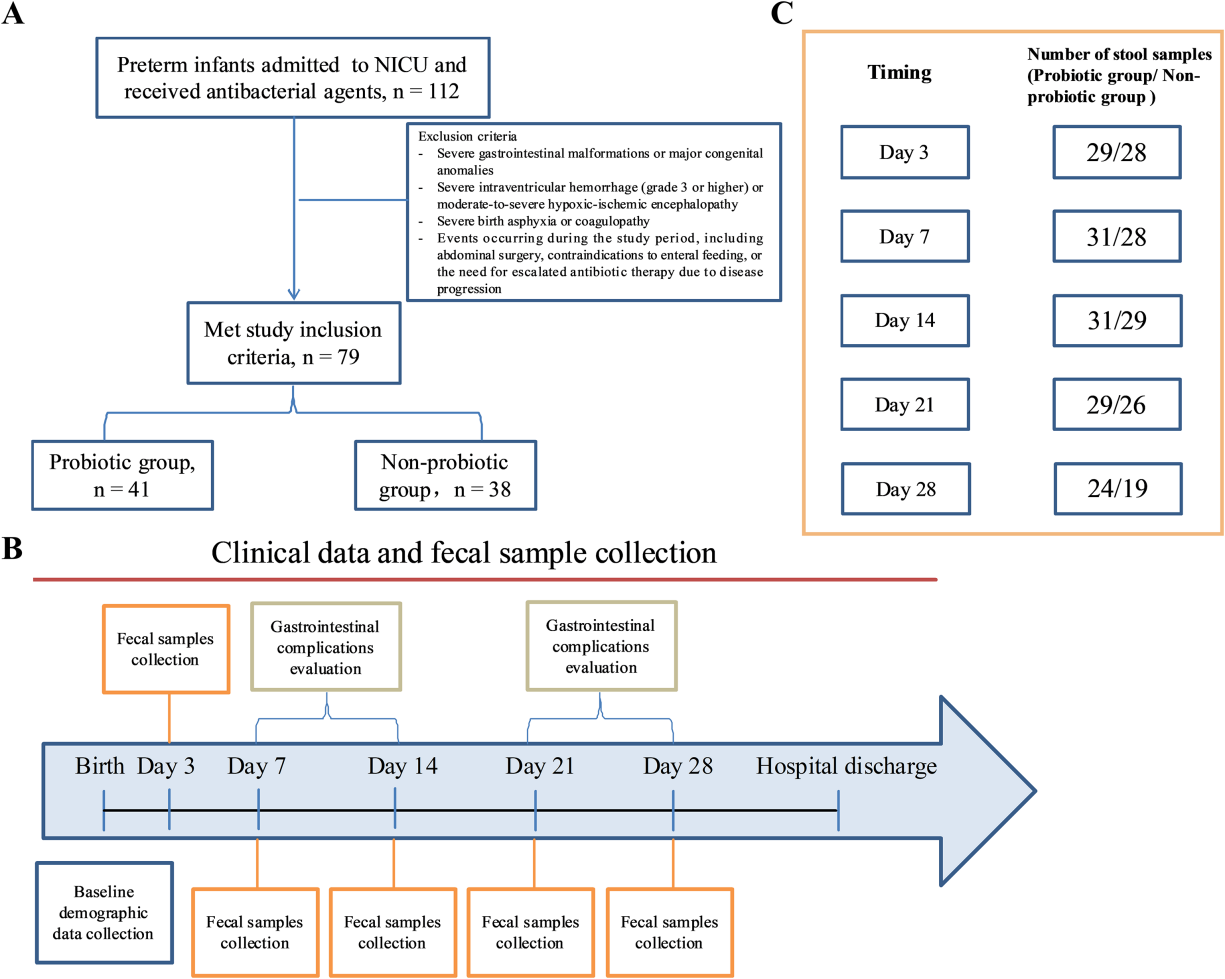
Study profile and sample collection. **(A)** Flowchart of participant recruitment and group allocation. **(B)** Schematic diagram of the enrollment period and longitudinal sampling time points. **(C)** Number of fecal samples collected at each time point for the probiotic and non-probiotic groups in the longitudinal microbiota analysis.

The study protocol was approved by the Medical Ethics Committee of the Women and Children’s Hospital of Ningbo University (approval No. EC2019-024), and written informed consent was obtained from the parents or legal guardians of all participants. A flowchart of the study recruitment process is presented in **Figure 1**.

### 2.2 Sample size and statistical power considerations

This was an exploratory observational cohort study. Due to the practical challenges of recruiting preterm infants in the real-world NICU setting, no *a priori* sample size estimation was performed; all consecutive eligible infants during the study period were enrolled (final n = 79: 41 probiotic, 38 non-probiotic). The minimum clinically important difference for the primary outcome was set at a 20% absolute reduction in FI incidence, in accordance with published clinical guidelines (Evidence-Based Medicine Group, 2020), prior clinical research in similar preterm infant populations (Soderquist Kruth et al., 2025), and our institutional clinical practice experience. A *post-hoc* minimum detectable effect size analysis confirmed that with two-sided α=0.05 and 80% power, the study could reliably detect an OR ≤ 0.30 or ≥ 3.33.

### 2.3 Clinical data collection

Baseline demographic and clinical characteristics were recorded for all infants, including gestational age, birth weight, sex, perinatal variables, and duration of invasive mechanical ventilation.

Feeding intolerance (FI), the primary clinical outcome, was defined by either of the following criteria: (i) gastric residual volume > 50% of the previous feeding volume, accompanied by vomiting and/or abdominal distension; or (ii) failure to advance enteral feeding as planned. To evaluate the temporal association between probiotic supplementation and FI, the 28-day observation period was stratified into three distinct phases: Period I (baseline, pre-LCBE: postnatal days 1–3), Period II (during LCBE administration: days 7–14), and Period III (post-LCBE: days 21–28). For each period, an infant was classified as having FI if they met the diagnostic criteria at any point within that window. Importantly, an FI event in an earlier period did not automatically classify an infant as having FI in subsequent periods.

Laboratory monitoring included longitudinal assessment of complete blood count (CBC) parameters and C-reactive protein (CRP). Age-specific normal reference ranges were defined as follows: for infants ≤ 7 days old, white blood cell (WBC) count: 15–20 × 10^9^/L, neutrophil count: 1.8–6.3 × 10^9^/L, eosinophil count: 0.02–0.52 × 10^9^/L, and CRP: 0–8 mg/L; for infants > 7 days old, the WBC range was adjusted to 5–12 × 10^9^/L, while the reference ranges for neutrophils, eosinophils, and CRP remained unchanged. An abnormal value was defined as a measurement exceeding the upper limit or falling below the lower limit of the corresponding reference range by more than 10%.

All clinical data were extracted from electronic medical records and analyzed anonymously.

### 2.4 Fecal sample collection

Fecal samples were collected from disposable diapers using sterile swabs on postnatal days 3, 7, 14, 21, and 28. Each sample (approximately 2 g) was immediately stored at −80 °C until further processing. To be included in the final microbiome analysis, infants were required to provide fecal samples of sufficient quantity from at least three of the five scheduled time points. A total of 274 samples met this criterion and were included in the analysis (144 from the probiotic group and 130 from the non-probiotic group; **Figure 1C**).

To minimize potential confounding by seasonal variations in gut microbiota establishment, sample collection was primarily concentrated in the cooler months: 230 samples (83.9%) were collected between December and March, and the remaining 44 (16.1%) were collected in April and May.

### 2.5 DNA extraction and 16S rRNA amplicon sequencing

Genomic DNA was extracted from fecal samples using the Tiangen Fecal DNA Extraction Kit (Tiangen Biochemical Technology Co., Ltd.) following the manufacturer’s protocol. The V3–V4 hypervariable region of the bacterial 16S rRNA gene was amplified using primers 338F (5′-ACTCCTACGGGAGGCAGCA-3′) and 806R (5′-GGACTACHVGGGTWTCTAAT-3′). Sequencing libraries were prepared using a PCR-free sample preparation kit with dual-index barcodes and quantified via Qubit fluorometry (Thermo Fisher Scientific). High-throughput sequencing was performed on the Illumina NovaSeq 6000 platform. Each sample was sequenced to a depth of ≥ 50, 000 valid reads.

### 2.6 Sequencing data analysis

Sequence data were processed using the Divisive Amplicon Denoising Algorithm 2 (DADA2) plugin in Quantitative Insights Into Microbial Ecology 2 (QIIME 2) for quality filtering, denoising, paired-end read merging, and chimera removal, generating amplicon sequence variants (ASVs). Microbiota analyses were performed using QIIME 2 and R packages. Alpha diversity indices (Shannon and Simpson) were calculated to assess microbial diversity and evenness. Functional profiling was inferred using the Phylogenetic Investigation of Communities by Reconstruction of Unobserved States 2 (PICRUSt2) pipeline, with ASVs mapped to the Kyoto Encyclopedia of Genes and Genomes (KEGG) Orthology (KO) database.

### 2.7 Statistical analysis

Categorical variables were compared using the chi-square test, and Fisher’s exact test was applied when expected cell counts were <5. Continuous variables were compared using the Wilcoxon rank-sum test. Longitudinal differential abundance analysis of gut microbiota was performed using the DESeq2 package in R. Subject ID was included in the statistical model to account for repeated measurements within individuals, and pairwise comparisons across the five time points were conducted separately within each group. Multivariate binary logistic regression was used to calculate adjusted odds ratios (OR) and 95% confidence intervals (CI) for the association between LCBE and FI. All statistical analyses were performed using SPSS version 25.0 (IBM, Armonk, NY, USA) and R statistical software (R Core Team, Vienna, Austria), with *p* < 0.05 considered statistically significant.

## 3 Results

### 3.1 Characteristics of the study participants

A total of 79 preterm infants (46 males, 33 females) were included: 41 in the probiotic group and 38 in the non-probiotic group, with a median gestational age of 30 + 0 weeks (IQR: 28 + 4–31 + 6) and a median birth weight of 1350 g (IQR: 1180–1550 g). Baseline characteristics were comparable between groups, except for birth weight, which was adjusted for in subsequent regression analyses (**Table 1**). No statistically significant differences were found between the groups in the duration of invasive mechanical ventilation during the study period (*p* > 0.05, **Table 1**). The duration of nasogastric tube feeding differed between groups (*p* = 0.015, **Table 1**), consistent with baseline differences in birth weight.

**Table 1:** Baseline clinical characteristics of the two groups of preterm infants.

Characteristic	Total (n=79)		
	Probiotic group, (n=41)	Non-probiotic group, (n=38)	*P* value
Sex (male/female), n	22/19	24/14	0.392
Gestational age, wk (M, P25–P75)	29+6 (28 + 3–31+0)	30+4 (28 + 6–32+4)	0.112
Birth weight, g (M, P25–P75)	1290 (1135–1450)	1525 (1207–1845)	0.012
Cesarean delivery, n (%)	23 (56.10%)	27 (71.05%)	0.168
Antenatal antibiotic exposure, n (%)	15 (36.59%)	18 (47.37%)	0.332
Asphyxia ^a^, n (%)	7 (17.07%)	4 (10.53%)	0.521
1-min Apgar score (M, P25–P75)	8.00 (8.00–8.00)	8.00 (8.00–9.00)	0.159
5-min Apgar score (M, P25–P75)	9.00 (9.00–9.00)	9.00 (9.00–10.00)	0.100
Premature rupture of membranes, n (%)	13 (31.71%)	15 (39.47%)	0.160
Invasive ventilator use time ^b^, d (M, P25–P75)	1 (0–8.5)	2 (0–4.25)	0.592
Duration of exclusive nasogastric tube feeding ^b^, d (M, P25–P75)	28 (28–28)	28 (25.75–28)	0.015

Data are presented as median (interquartile range [IQR]).

^a^ All cases were mild asphyxia.

^b^ Collected during days 1–28 after birth.

### 3.2 In-hospital clinical and laboratory outcomes

In both groups, the incidence of FI increased from baseline to the during-supplementation period and then declined (**Table 2**). Period-specific analysis revealed no significant difference during Period I (pre-LCBE) or Period II (during LCBE). However, during Period III (post-LCBE, days 21–28), the non-probiotic group had a significantly higher incidence of FI than the probiotic group (44.74% vs. 17.07%; adjusted OR = 0.156, 95% CI: 0.050–0.491, *p* = 0.001; **Table 2**). This corresponds to an 84.4% relative reduction in the independent risk of FI associated with LCBE supplementation. Importantly, this protective association became evident only after the 14-day course had been completed, indicating a temporal delay in the clinical benefit. No drug-related adverse events were reported.

**Table 2:** Clinical outcomes of the two groups of preterm infants.

Outcome	Probiotic group (n=41)	Non-probiotic group (n=38)	Adjusted OR (95% CI)*	*P* value
FI ^a^, n (%)	5 (12.20)	5 (13.16)	—	0.898
FI ^b^, n (%)	14 (34.15)	18 (47.37)	0.380 (0.139-1.036)	0.059
FI ^c^, n (%)	7 (17.07)	17 (44.74)	0.156 (0.050-0.491)	0.002

FI, Feeding intolerance; CI, confidence interval.

^a^ Collected during days 1–3 after birth.

^b^ Collected during days 7–14 after birth.

^c^ Collected during days 21–28 after birth.

*Adjusted for birth weight.

**Figure 2:**
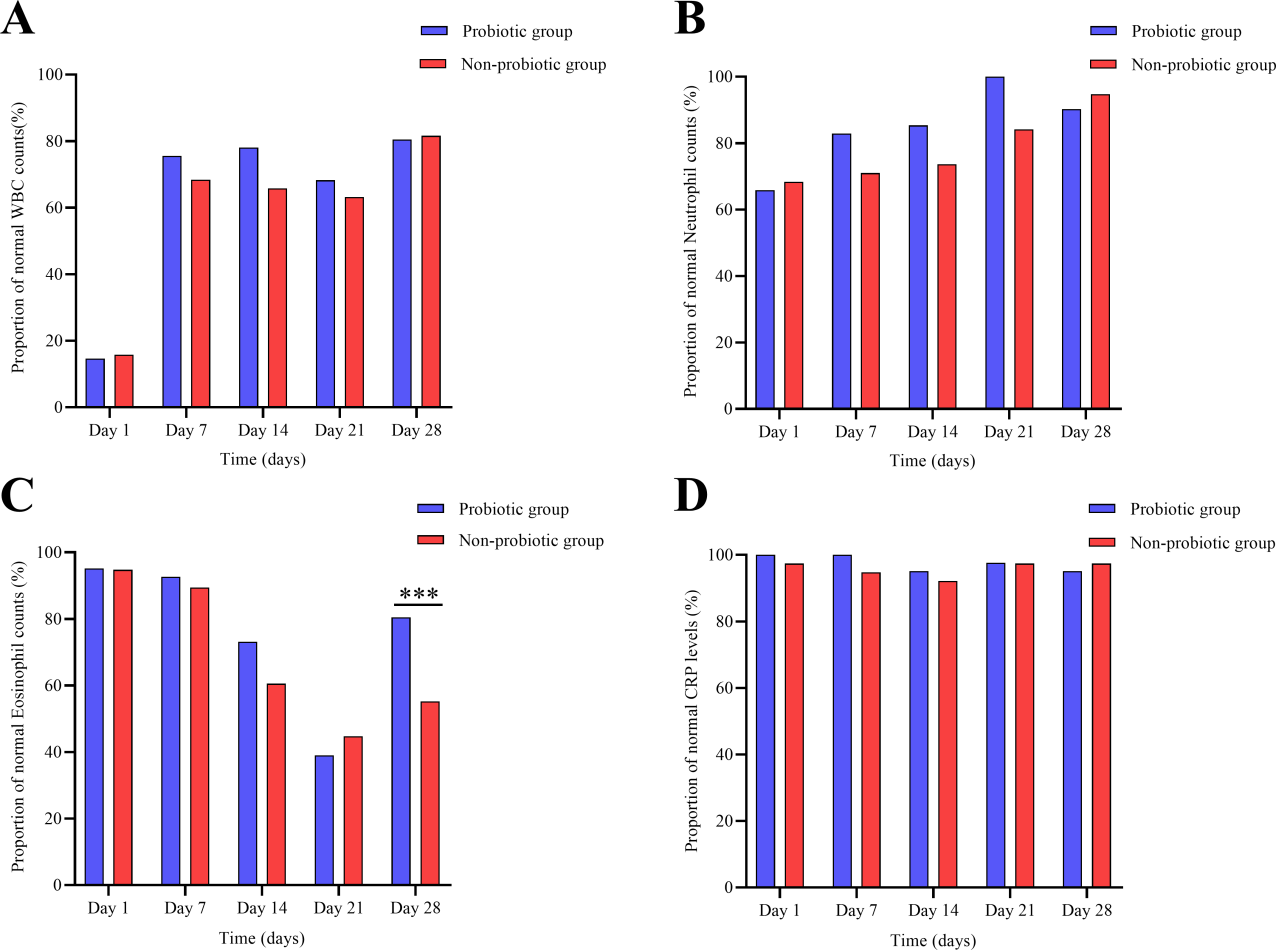
Longitudinal assessment of routine blood parameters. The proportion of infants with **(A)** white blood cell (WBC) counts, **(B)** neutrophil counts, **(C)** eosinophil counts, and **(D)** C-reactive protein (CRP) levels within the normal reference range at postnatal days 1, 7, 14, 21, and 28. Normal ranges were age-specific for WBC: 15–20 × 10^9^/L for infants ≤ 7 days old, and 5–12 × 10^9^/L for infants > 7 days old. Reference ranges for neutrophils (1.8–6.3 × 10^9^/L), eosinophils (0.02–0.52 × 10^9^/L), and CRP (0–8 mg/L) were consistent across all ages. ***p < 0.001 for comparison between groups.

Routine blood parameters, including WBC counts, neutrophil counts, and C-reactive protein (CRP) levels, were monitored longitudinally, and no significant differences were observed between the two groups at any time point (**Figure 2**). However, a notable difference was observed for eosinophil counts. As shown in **Figure 2C**, while the proportion of infants with eosinophil counts within the normal range was comparable between groups during the first three weeks, a significant divergence emerged on postnatal day 28. On day 28, the proportion of infants with eosinophil counts exceeding the upper limit of normal was significantly higher in the non-probiotic group compared to the probiotic group (*p* < 0.001).

### 3.3 Association between LCBE supplementation and gut microbiota

**Figure 3:**
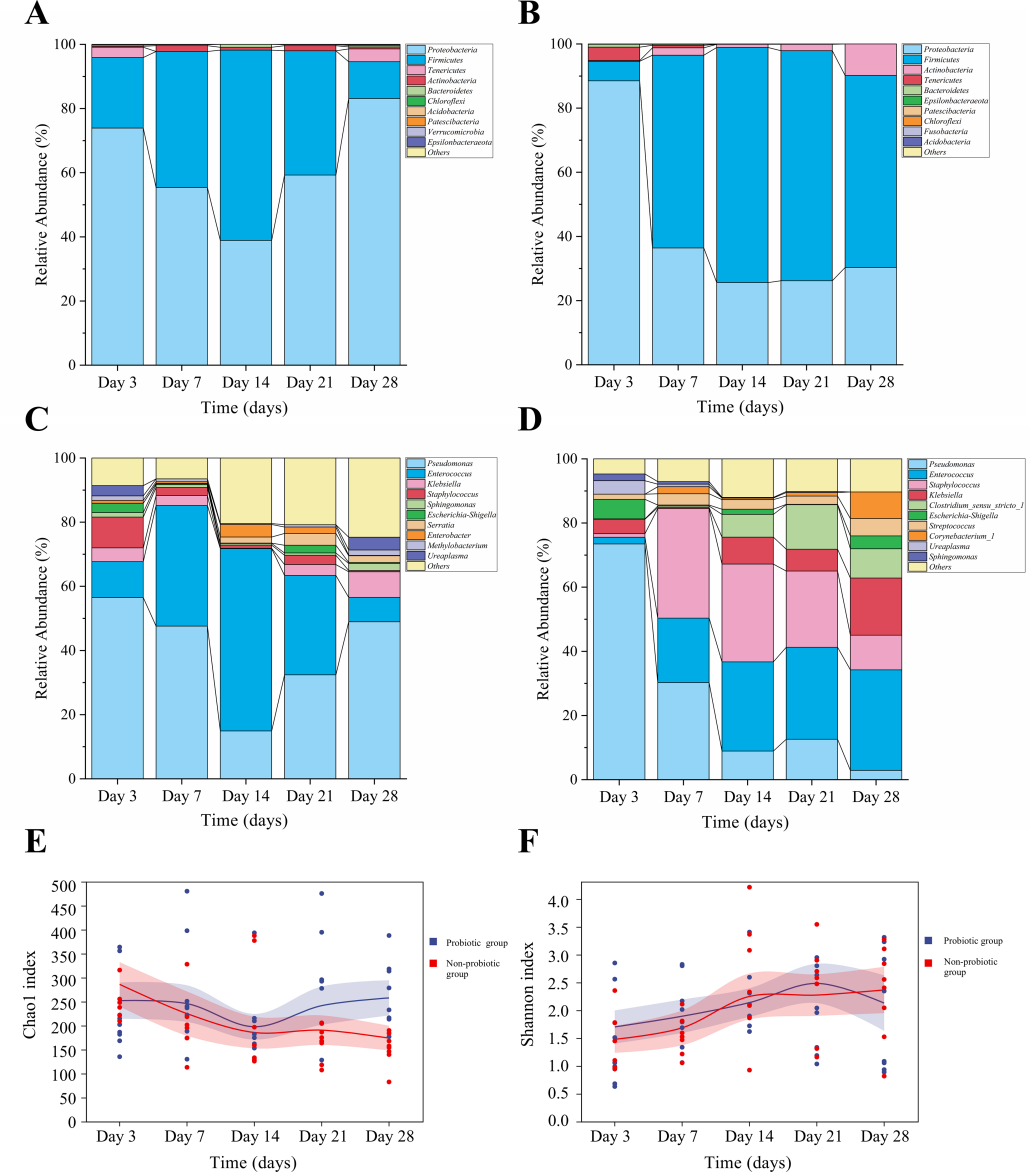
Longitudinal dynamics of gut microbiota composition and diversity. Relative abundance of bacterial taxa at the phylum level in the **(A)** probiotic and **(B)** non-probiotic groups. Relative abundance at the genus level in the **(C)** probiotic and **(D)** non-probiotic groups. Alpha diversity metrics over time: **(E)** Chao1 richness index and **(F)** Shannon diversity index. Data show temporal trends within each group.

16S rRNA sequencing revealed distinct differences in gut microbiota composition between the groups. To improve clarity and conciseness, we combined the compositional and diversity results in a single figure (**Figure 3**). At the phylum level, the five most dominant phyla (Proteobacteria, Firmicutes, Tenericutes, Actinobacteria, and Bacteroidetes) exhibited distinct temporal dynamics between groups (**Figures 3A, B**). At the genus level, *Pseudomonas* displayed the highest average relative abundance, followed by *Enterococcus*, and both genera showed divergent temporal dynamics between the two groups (**Figures 3C, D**). Regarding *α*-diversity, the Chao1 index in the probiotic group declined initially but then increased from days 14 to 28, whereas the non-probiotic group showed a steady decline (**Figure 3E**). The Shannon index trended upward in both groups, with a slight dip in the probiotic group on day 28 (**Figure 3F**).

**Figure 4:**
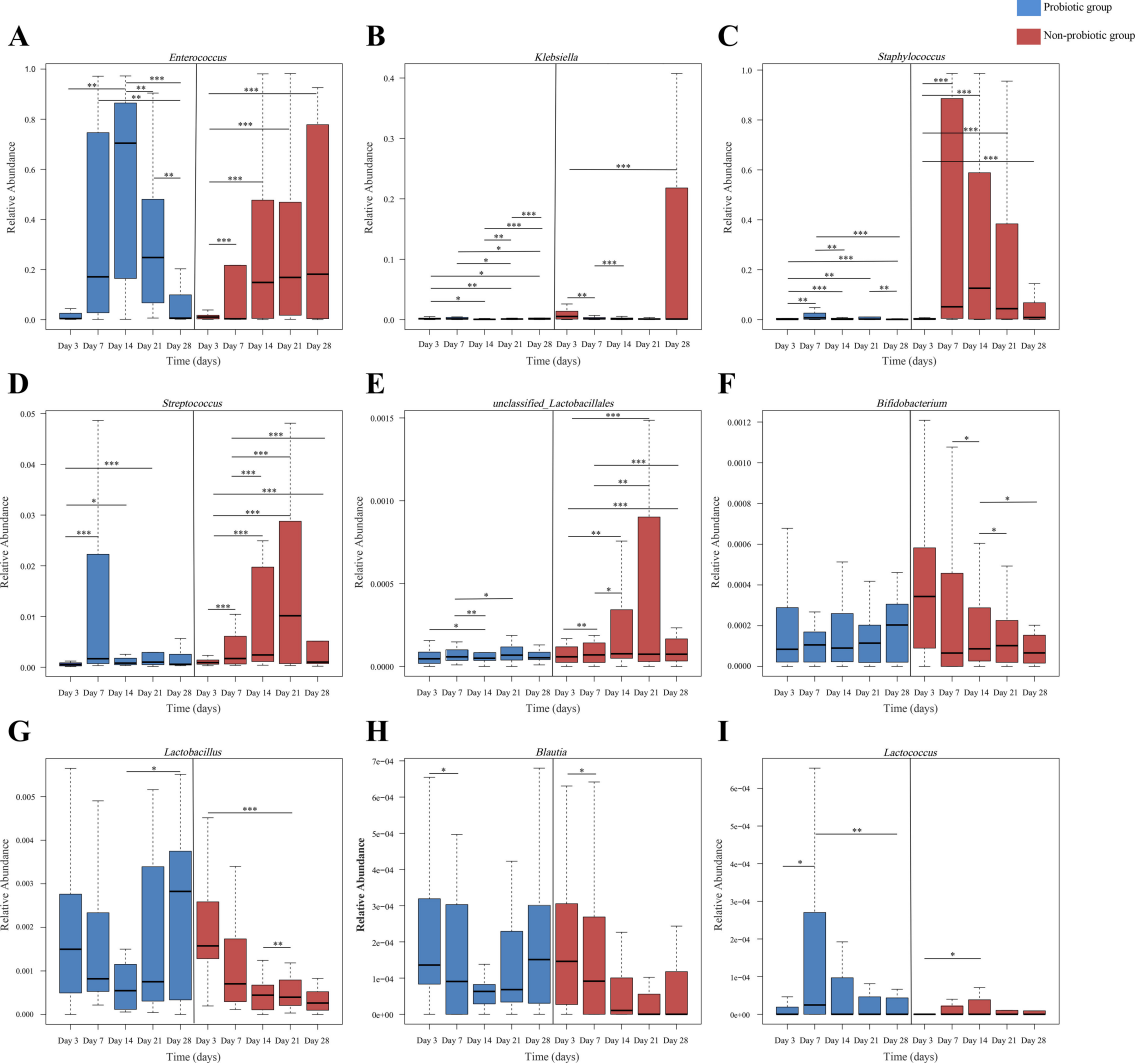
Temporal changes in the relative abundance of key genera. Comparison of genus-level relative abundances between the probiotic and non-probiotic groups over the 28-day study period for **(A)** *Enterococcus*, **(B)** *Klebsiella*, **(C)** *Staphylococcus*, **(D)** *Streptococcus*, **(E)** *unclassified Lactobacillales*, **(F)** *Bifidobacterium*, **(G)** *Lactobacillus*, **(H)** *Blautia*, and **(I)** *Lactococcus*. *p < 0.05, **p < 0.01, ***p < 0.001. Intra-group temporal trends are shown.

Differential abundance analysis identified genera with significant group-specific trends (**Figure 4**). In the non-probiotic group, *Enterococcus*, *Klebsiella*, *Staphylococcus*, *Streptococcus*, and *unclassified Lactobacillales* exhibited significant increases in relative abundance (*p <* 0.01) by day 21 or 28, whereas these taxa either decreased or remained stable in the probiotic group (**Figures 4A–E**). Conversely, *Bifidobacterium* and *Lactobacillus* showed increasing or stable relative abundances in the probiotic group but declined significantly in the non-probiotic group (**Figures 4F, G**). To explore whether these compositional shifts mediated the protective effect of LCBE against FI, we performed Spearman correlation analyses between the relative abundances of the differentially abundant genera and FI incidence. No statistically significant linear associations were detected for any single genus (all *p* > 0.05).

**Figure 5:**
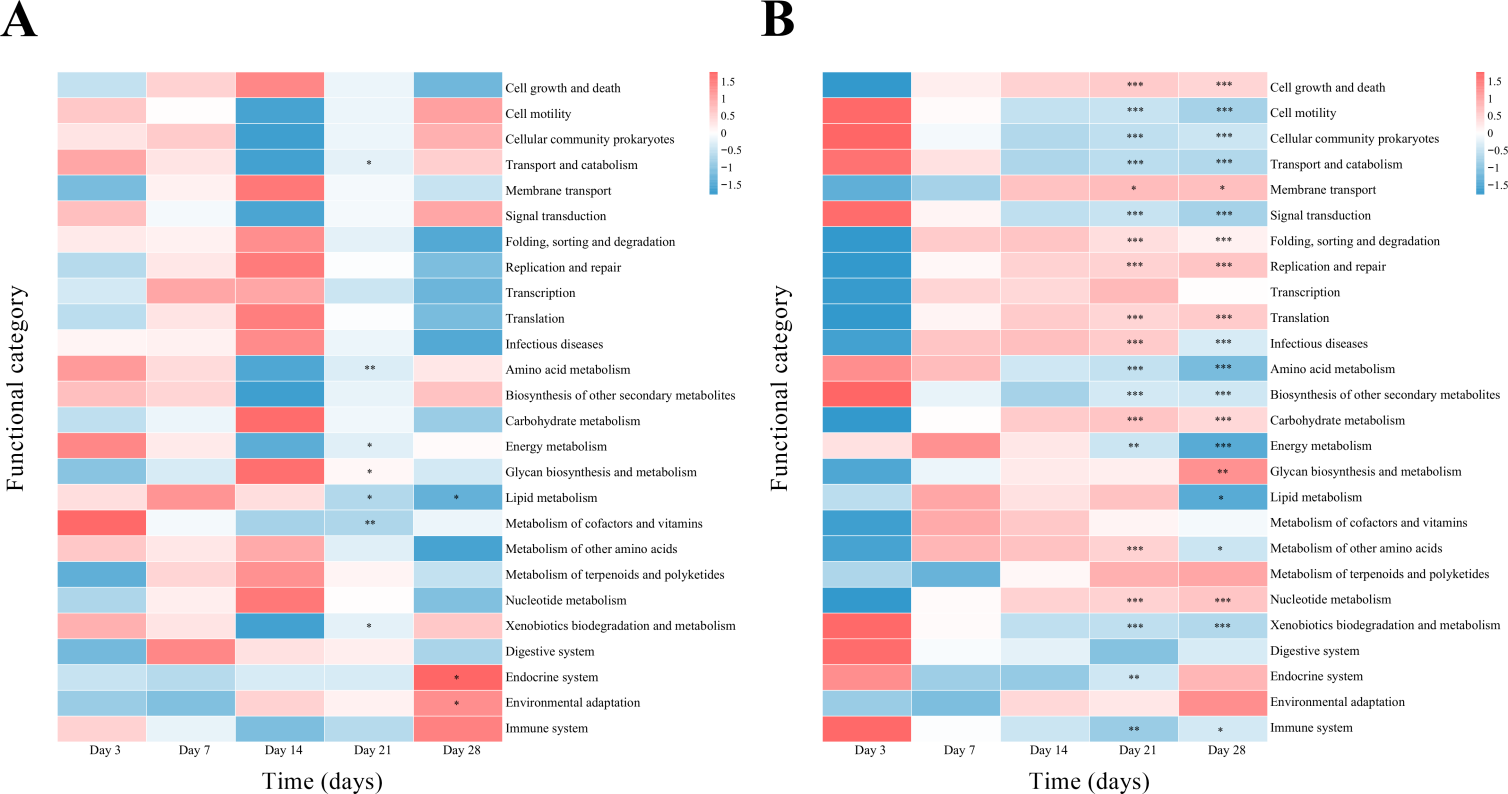
PICRUSt2-predicted functional profiles of the gut microbiota. Heatmaps showing predicted secondary-level KEGG pathways in the **(A)** probiotic and **(B)** non-probiotic groups across five time points. Within-group comparisons (day 3 vs. days 21/28): *p < 0.05, **p < 0.01, ***p < 0.001. PICRUSt2, Phylogenetic Investigation of Communities by Reconstruction of Unobserved States 2; KEGG, Kyoto Encyclopedia of Genes and Genomes.

Notably, PICRUSt2-predicted functional profiles diverged significantly between the probiotic and non-probiotic groups from postnatal days 21 to 28 (**Figure 5**). In the probiotic group, pathways related to infectious diseases were downregulated, while those involved in the immune system, metabolism, and signal transduction were upregulated or stabilized. The opposite pattern was observed in the non-probiotic group. These predictive data suggest that LCBE may support the maturation of microbial metabolic functions, although direct metagenomic confirmation is needed.

## 4 Discussion

The core finding of this study is that early LCBE supplementation in antibiotic-exposed preterm infants was independently associated with a significantly lower incidence of FI during postnatal days 21–28 (adjusted OR = 0.156, 95% CI: 0.050–0.491, *p* = 0.001), corresponding to an 84.4% relative risk reduction. This clinical benefit aligns with the established paradigm that antibiotic-induced gut dysbiosis represents an important contributing factor to gastrointestinal complications in preterm infants, and thus provides direct clinical evidence supporting the utility of probiotic intervention for improving feeding tolerance in this high-risk population. This is consistent with previous clinical consensus guidelines and systematic reviews (Evidence-Based Medicine Group, 2020; Soderquist Kruth et al., 2025), further confirming the rationality of our intervention strategy and the robustness of the study findings. Critically, the protective effect emerged only after the 14-day LCBE course had been completed, indicating a delayed yet sustained benefit. This temporal pattern is a key finding, as it suggests that LCBE does not act as a direct pharmacological agent but rather initiates a process of microbiota reconstitution that continues to evolve even after the intervention is withdrawn. This finding is clinically significant, as FI not only impairs nutritional intake and growth in preterm infants and prolongs hospitalization, but also serves as an important precursor to NEC (Alqahtani, 2025). By demonstrating a significant reduction in FI, our study suggests that LCBE supplementation may confer clinical benefits that extend beyond symptom improvement to potentially mitigate the risk of more severe neonatal outcomes.

The intestinal microbiota is critical for neonatal health, but antibiotics disrupt its composition and function, predisposing infants to complications such as gastrointestinal dysfunction (Navarro-Tapia et al., 2020; Aguilar-Lopez et al., 2021). As noted in the introduction, early antibiotic exposure has been shown to disrupt gut microbiota colonization and diversity, with particularly marked impacts on beneficial genera such as *Bifidobacterium* and *Lactobacillus* (Tanaka et al., 2009; Fouhy et al., 2012). Previous studies have shown that early probiotic supplementation may mitigate the deleterious effects of antibiotics and cesarean delivery, improving feeding tolerance and reducing associated complications (Mitha et al., 2022; Sowden et al., 2022). In this cohort, we found that LCBE supplementation was associated with significant modulation of the gut microbiota, including suppression of opportunistic pathogens (e.g., *Enterococcus*, *Klebsiella*) and preservation of beneficial commensals (e.g., *Bifidobacterium*, *Lactobacillus*). These microbial changes were accompanied by a more stable α-diversity trajectory and predicted functional maturation in the probiotic group, whereas the non-probiotic group exhibited progressive dysbiosis and enrichment of pathogenic functions.

Although we did not detect a significant linear correlation between the abundance of any single genus and FI incidence, we interpret this finding with caution. FI is a multifactorial clinical syndrome, and its improvement may depend on the collective behavior of the microbial community rather than on individual taxa (Ford et al., 2019; Marsubrin et al., 2023; Shao et al., 2024). Indeed, higher gut microbial diversity—reflecting a more resilient community structure—has been associated with reduced FI in preterm infants (Ford et al., 2019), whereas attempts to attribute FI to single taxa or simple ratios have yielded negative results (Marsubrin et al., 2023). Recent ecological evidence further supports that neonatal microbiota assembly is determined by overall community states, which dictate long-term functional outcomes such as pathogen resistance (Shao et al., 2024). The overall shift toward a more resilient and mature microbiota—characterized by a reduced pathogen load and preserved beneficial bacteria—likely contributes to enhanced intestinal barrier function and immune homeostasis, thereby lowering the risk of FI. Notably, the enrichment of opportunistic pathogens (e.g., *Klebsiella*) observed in the non-probiotic group has been associated with NEC in other studies (Devarajalu et al., 2025). Thus, the microbial modulation associated with LCBE supplementation may confer protection not only against FI but also against the downstream risk of these more severe complications.

Our observations provide microbiological support for the hypothesis that LCBE may exert its clinical effects through holistic ecosystem modulation. The delayed emergence of the clinical benefit (post-supplementation) is consistent with this ecological interpretation. Building on the synergistic mechanisms of LCBE outlined in the introduction—whereby *B. subtilis* reduces intestinal oxygen tension to support beneficial anaerobes and *E. faecium* reinforces intestinal homeostasis—we propose that LCBE could act as an ecological “pioneer species” modulator, a concept aligned with the theory of priority effects in microbial community assembly (Sprockett et al., 2018). By transiently occupying niches and potentially reducing intestinal oxygen tension (via *B. subtilis*), it may create a permissive environment for the establishment of a more stable and beneficial microbial community. This newly assembled community, once established, might contribute to pathogen resistance through complementary mechanisms, including competitive exclusion (Yin et al., 2025) and the production of inhibitory metabolites such as short-chain fatty acids (SCFAs) (Mann et al., 2024). Such effects could persist even after discontinuation of the probiotic supplement, consistent with ecological models of community assembly (Sprockett et al., 2018). This community-based resistance, if confirmed, would represent a more sustainable mechanism than a direct transient effect of the probiotic itself—which could explain the observed temporal lag in clinical benefit. This framework also offers a plausible explanation for why no single taxon correlated linearly with FI; the putative benefit may arise from emergent properties of the entire network rather than from any single member. Our findings align with and extend the recent work by Kiu et al., who demonstrated that probiotic supplementation in preterm infants can reduce the burden of antibiotic resistance genes and restore an age-appropriate microbiota (Kiu et al., 2025). While their work focused on genomic and resistome-level outcomes, our study provides a clinical correlate: this probiotic-associated ecological shift was accompanied by a significantly lower incidence of FI in our cohort. Together, these complementary studies suggest that probiotics may confer multifaceted benefits in this vulnerable population, linking microbial ecology with patient outcomes.

We also observed that by postnatal day 28, the non-probiotic group had a significantly higher proportion of infants with elevated eosinophil counts (*p* < 0.001). Elevated eosinophils are a marker of allergic or inflammatory processes (Jackson et al., 2022). Probiotics may inhibit excessive eosinophil activation via microbiota modulation (Han et al., 2012; Lu et al., 2025), raising the possibility of an additional anti-inflammatory effect of LCBE in this population.

The functional predictions from PICRUSt2, while preliminary, indicated that LCBE was associated with downregulation of infectious disease-related pathways and upregulation of pathways involved in immunity, metabolism, and signal transduction during days 21–28. These functional shifts are consistent with the observed clinical and taxonomic changes. The downregulation of pathogenicity-related functions in the probiotic group provides a functional rationale for the suppression of opportunistic pathogens like *Klebsiella* and *Enterococcus*. Conversely, the upregulation or stabilization of metabolic and immune pathways aligns with the preservation of beneficial genera like *Bifidobacterium* and *Lactobacillus*, which are key contributors to these functions (Peng et al., 2020; Chi et al., 2021; Brown et al., 2023). This congruence between taxonomic composition, predicted function, and clinical outcome strengthens our hypothesis that LCBE fosters a gut ecosystem that is not only structurally different but also functionally more mature and host-supportive. However, because these are predictive inferences based on 16S data, they require validation through metagenomic or metabolomic analyses in future studies.

This study has several limitations. First, as a single-center observational study with a modest sample size (n = 79), causal inference cannot be established. Although the study was adequately powered to detect the large effect of LCBE on FI, it may be underpowered for detecting weak-to-moderate correlations between individual taxa and clinical outcomes, and intermittent missing fecal samples across longitudinal time points further slightly reduced the sample size of pairwise temporal comparisons of gut microbiota. Second, the use of PICRUSt2 provides only predicted functional profiles, which require validation through metagenomic or metabolomic analyses. Third, the follow-up was limited to 28 days, and although we adjusted for birth weight, residual confounding cannot be completely ruled out. Fourth, our cohort was restricted to exclusively formula-fed infants to minimize confounding, which may limit the generalizability of our findings to breastfed preterm infants. Future studies should evaluate the efficacy of LCBE in breastfed populations. Despite these limitations, this study provides the first preliminary evidence from a well-defined cohort with paired longitudinal microbial data, demonstrating an association between LCBE and both clinical outcomes and gut microbiota composition in antibiotic-exposed preterm infants. By proposing an ecological mechanism—whereby “pioneer species” modulation may contribute to sustained community-based protection—our findings help address an important knowledge gap in NICU pharmaceutical care and provide a clear scientific basis for subsequent multicenter randomized controlled trials to confirm efficacy and explore these mechanistic pathways.

## 5 Conclusions

In this study, early LCBE supplementation in antibiotic-exposed preterm infants was associated with a significantly lower incidence of FI during the post-supplementation period (days 21–28). This clinical benefit was accompanied by favorable modulation of the gut microbiota, including the suppression of opportunistic pathogen overgrowth and the preservation of beneficial commensal taxa, and was associated with more stable microbial diversity with evidence of functional maturation. These findings suggest that LCBE is safe and may be a promising adjunctive strategy in this high-risk population. This preliminary evidence provides a strong rationale for prioritizing larger, multicenter randomized controlled trials to confirm its efficacy and long-term safety.

## Data Availability

The datasets presented in this study can be found in online repositories. The names of the repository/repositories and accession number(s) can be found below: https://www.ncbi.nlm.nih.gov/, PRJNA1304471.
